# A20 prevents chronic liver inflammation and cancer by protecting hepatocytes from death

**DOI:** 10.1038/cddis.2016.154

**Published:** 2016-06-02

**Authors:** L Catrysse, M Farhang Ghahremani, L Vereecke, S A Youssef, C Mc Guire, M Sze, A Weber, M Heikenwalder, A de Bruin, R Beyaert, G van Loo

**Affiliations:** 1 Inflammation Research Center, VIB, Ghent B-9052, Belgium; 2 Department of Biomedical Molecular Biology, Ghent University, Ghent B-9052, Belgium; 3 Dutch Molecular Pathology Center, Department of Pathobiology, Faculty of Veterinary Medicine, Utrecht University, Utrecht NL-3584, The Netherlands; 4 Institute of Surgical Pathology, University Zurich, Zurich CH-8091, Switzerland; 5 Institute of Virology, Technische Universität München, Munich D-81675, Germany; 6 Division of Chronic Inflammation and Cancer, German Cancer Research Center, Heidelberg D-69120, Germany; 7 University Medical Center Groningen, Department of Pediatrics, University of Groningen, Groningen NL-9713, The Netherlands

## Abstract

An important regulator of inflammatory signalling is the ubiquitin-editing protein A20 that acts as a break on nuclear factor-*κB* (NF-*κB*) activation, but also exerts important cytoprotective functions. A20 knockout mice are cachectic and die prematurely due to excessive multi-organ inflammation. To establish the importance of A20 in liver homeostasis and pathology, we developed a novel mouse line lacking A20 specifically in liver parenchymal cells. These mice spontaneously develop chronic liver inflammation but no fibrosis or hepatocellular carcinomas, illustrating an important role for A20 in normal liver tissue homeostasis. Hepatocyte-specific A20 knockout mice show sustained NF-*κB*-dependent gene expression in the liver upon tumor necrosis factor (TNF) or lipopolysaccharide injection, as well as hepatocyte apoptosis and lethality upon challenge with sublethal doses of TNF, demonstrating an essential role for A20 in the protection of mice against acute liver failure. Finally, chronic liver inflammation and enhanced hepatocyte apoptosis in hepatocyte-specific A20 knockout mice was associated with increased susceptibility to chemically or high fat-diet-induced hepatocellular carcinoma development. Together, these studies establish A20 as a crucial hepatoprotective factor.

Apoptotic cell death plays an important role in liver disease, and tumor necrosis factor receptor-1 (TNFR1) and Fas (CD95) are the most prominent cell death receptors involved.^1^ Both TNF and Fas ligand initiate a signalling pathway leading to apoptosis through receptor Fas-associated death domain (FADD) recruitment and activation of procaspase-8 leading to downstream procaspase-3 activation. TNF is distinct from Fas signalling as it first activates nuclear factor-*κB* (NF-*κB*) and c-jun N-terminal kinase(JNK).^2^ The transcription factor NF-*κB* controls cytoprotective activities by inducing the expression of important anti-apoptotic genes including A20, Bcl-xL and c-FLIP, and through inhibition of prolonged JNK activation.^3^ However, when protective NF-*κB* activation is compromised, the activated TNF receptor complex will induce the activation of an apoptotic cascade.^2^ The involvement of TNF and Fas signalling in liver apoptosis was previously confirmed in transgenic mice overexpressing a dominant-negative FADD mutant, and in hepatocyte-specific caspase-8 deficient mice, showing a complete rescue from TNF- and Fas-induced liver failure.^4, 5, 6^ Besides apoptosis, which is generally considered a non-inflammatory cell death mode due to rapid clearance of apoptotic cells by phagocytes, TNF can also induce necroptosis, which is characterized by cellular swelling and membrane leakage, inducing strong inflammatory responses and thus highly relevant for many types of liver diseases.^1^

Next to acute liver failure, which can be caused by hepatotoxins, foodborne poisons, alcohol intoxication or infections, and is characterized by massive hepatocyte apoptosis associated with life-threatening consequences, most liver pathologies result from chronic disease processes involving inflammation, continuous hepatocyte apoptosis and compensatory tissue regeneration, promoting the development of liver fibrosis, cirrhosis and eventually hepatocellular carcinoma (HCC). Many of the inflammatory mediators involved in these liver diseases are targets or activators of NF-*κB*. However, besides its crucial contribution to these detrimental inflammatory reactions in liver, NF-*κB* also contributes to liver homeostasis and wound-healing processes.^7^

The NF-*κB* responsive and ubiquitin-editing protein A20 (also referred to as TNF alpha-induced protein 3 or TNFAIP3) is essential for the termination of NF-*κB* signalling in response to TNF and microbial products such as lipopolysaccharide (LPS) and muramyl dipeptide,^8, 9^ but also negatively regulates TNF-induced apoptosis.^10, 11^ Interestingly, A20 has been identified as a susceptibility locus for multiple immunopathologies,^12^ including autoimmune hepatitis.^13^ Using A20 heterozygous mice, or mice that transiently overexpress an A20 cDNA, A20 in liver has been shown to be contributing to liver regeneration after partial hepatectomy^14, 15, 16, 17^ and acute toxic hepatitis^18^ through combined anti-apoptotic, anti-inflammatory and pro-proliferative functions.^19^

To study the role of endogenously expressed A20 in liver development and conditions of liver inflammation and hepatocarcinogenesis, we generated hepatocyte-specific A20 knockout mice. Here we show that, consistent with its role in NF-*κB* signalling, hepatocyte-specific A20 deficiency sensitizes mice to develop spontaneous liver inflammation, demonstrating a physiological role for A20 in regulating liver immune homeostasis. In agreement, A20 deficient hepatocytes display sustained NF-*κB* signalling and gene expression upon TNF or LPS challenge. Hepatocyte-specific A20 knockout mice are also hypersensitive to TNF-induced hepatocyte apoptosis and lethality, demonstrating an important cytoprotective function for A20 in hepatocytes. Finally, chronic liver inflammation and hepatocyte apoptosis sensitizes liver parenchymal cell-specific A20 knockout mice to the development of HCC in experimental models.

## Results

### Generation of mice lacking A20 specifically in liver parenchymal cells

**Figure 1:**
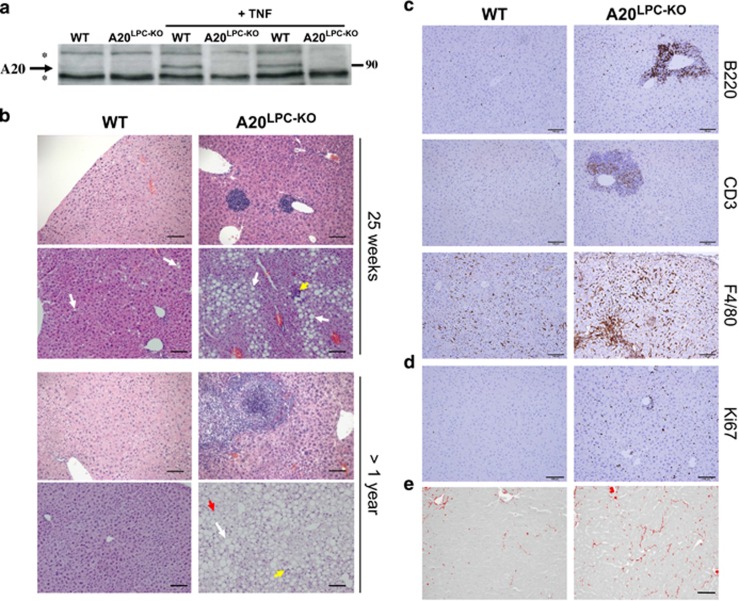
Molecular and histological analysis of hepatocyte-specific A20 knockout mice. (**a**) Western blot analysis for A20 expression in liver extracts from individual hepatocyte-specific A20 knockout (LPC-KO) and control WT littermate mice, either or not injected with recombinant mouse TNF. *, unspecific. (**b**) Representative pictures from hematoxylin and eosin-stained liver sections from hepatocyte-specific A20 knockout (A20^LPC-KO^) and control WT littermate mice. White arrow: Macrovesicular steatosis, yellow arrow: inflammatory foci, red arrow: balloon cell. (**c**) Immunostaining for infiltrating B cells (B220), T cells (CD3) and macrophages (F4/80), and (**d**) Ki67 staining for proliferating cells. (**e**) Representative picture of Picro Sirius red-stained sections from A20^LPC-KO^ and control WT littermate mice. Scale bar=100 *μ*m. WT, wild type

Mice with a conditional A20 allele, in which exons IV and V of A20 are flanked with two LoxP sites, were generated as described.^11^ In order to study the role of A20 in hepatocytes, we crossed the A20^FL/FL^ mice with a transgenic mouse line that expresses Cre under the control of the liver-specific albumin/alpha-fetoprotein promoter/enhancer (Alfp-Cre) and mediates efficient Cre recombination in liver parenchymal cells^20, 21^ (Supplementary Fig. S1). Hepatocyte-specific A20 knockout (A20^FL/FL^/Alfp-Cre, liver parenchymal cell-specific A20 knockout, A20^LPC-KO^) mice were born with normal Mendelian segregation and reached adulthood without any evidence of hepatic defects. Immunoblot analysis of liver protein extracts revealed efficient ablation of A20 in livers of A20^LPC-KO^ mice (Figure 1a).

### Phenotype of hepatocyte A20 deficient mice

A20^LPC-KO^ mice appear healthy and phenotypic analysis up to the age of 18 months revealed no obvious pathologies. Macroscopic dissection of livers from A20^LPC-KO^ mice revealed normal tissue morphology with absence of inflammatory foci, nodules or tumors. However, histological examination of liver sections from 25-week-old A20^LPC-KO^ mice revealed clear signs of chronic hepatitis and steatosis (Figure 1b, Table 1), and increased B, T cell and macrophage infiltration as demonstrated by anti-B220, -CD3 and –F4/80 immunohistochemistry, respectively (Figure 1c, Supplementary Fig. S2). Also, hepatocyte proliferation, as assessed by Ki67 staining, was strongly increased in livers from A20^LPC-KO^ mice, compared with control mice (Figure 1d). In old mice (>1 year of age), the severity of lobular inflammation, numbers of inflammatory foci and non-alcoholic fatty liver disease (NAFLD) activity (NAS) score (sum of steatosis+ballooning degeneration+inflammation) were significantly increased in the A20^LPC-KO^ mice when compared with the control group (Figure 1b, Table 1). No evidence of spontaneous fibrosis, as assessed by Picro Sirius red staining (Figure 1e, Table 1), or neoplasia was present in any of the examined mice. Taken together, these results demonstrate that conditional deletion of A20 in parenchymal liver cells results in a spontaneous mild to moderate liver inflammation and steatosis, however, without development of fibrosis or HCCs even at old age.

**Figure 2:**
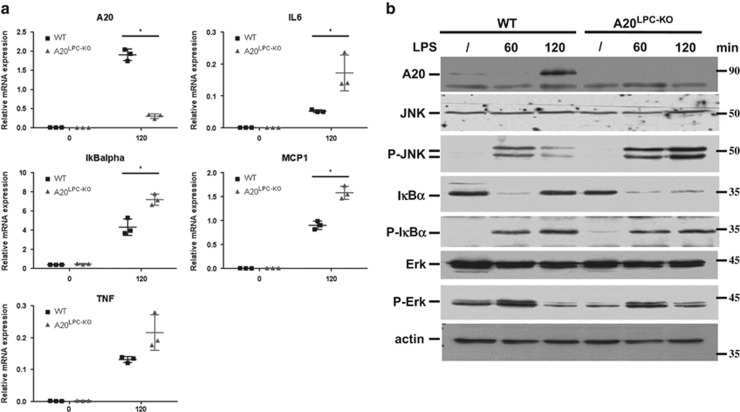
Increased cytokine expression, NF-*κB* activation and JNK activation in A20^LPC-KO^ mice. (**a**) Quantitative RT-PCR analysis of NF-*κB* response gene mRNA levels in the liver of 8-week-old A20^LPC-KO^ (*n*=3) and WT (*n*=3) littermate mice 120 min after PBS or LPS injection. Expression levels were normalized to reference genes HPRT and GAPDH using qbase+ software. Results are expressed as mean±s.d. *,*P*<0.05. (**b**) Liver protein extracts from 8-week-old LPS-injected A20^LPC-KO^ and WT mice subjected to immunoblot using antibodies detecting A20, JNK, phospho-JNK, I*κBα*, phospho-I*κBα*, Erk, phospho-Erk and actin. JNK, c-jun N-terminal kinase; LPS, lipopolysaccharide; WT, wild type

We next challenged A20^LPC-KO^ mice and control littermates with low dose LPS or TNF by intraperitoneal (i.p.) injection, and analysed their livers for altered inflammatory responses. In contrast to TNF that directly activates hepatocytes through binding to its receptor expressed on these cells, LPS can indirectly induce TNF release by Kupffer cells through binding to TLR4 expressed on these cells. Consistent with A20's role as an inhibitor of NF-*κB*-dependent gene expression, A20-deficient liver tissue displayed sustained degradation of the NF-*κB* inhibitory protein I*κBα* and significantly higher mRNA expression of *TNF, IL6, IκBα* and *MCP1* in response to LPS than wild-type cells (Figures 2a and b). Next to the hyperactivation of NF-*κB* signalling, analysis of other signalling events also showed enhanced JNK phosphorylation in LPS-treated A20^LPC-KO^ mice compared with wild-type controls (Figure 2b). The levels of phosphorylated ERK, however, were not different between both mouse lines (Figure 2b). Similar observations were made in mice challenged with low dose TNF injection (Supplementary Fig. S3).

Together, these results demonstrate a physiological role for A20 in restricting pro-inflammatory signalling in the liver.

### A20 protects hepatocytes from apoptosis in experimental hepatitis

**Figure 3:**
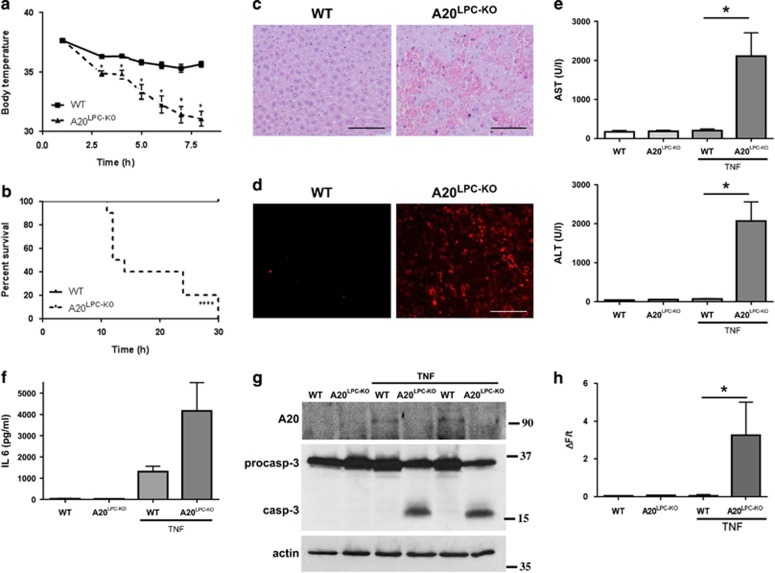
A20 protects hepatocytes from apoptosis in experimental hepatitis. (**a-b**) Body temperature (**a**) and survival (**b**) of A20^LPC-KO^ mice (*n*=10) and control (WT; *n*=10) littermates after i.p. injection of 10 μg mTNF. *,*P*<0.05; ****,*P*<0.0001 versus control. (**c**) Hematoxylin and eosin (H/E) staining on liver sections from A20^LPC-KO^ and control WT littermate mice injected with TNF for 5 h. (**d**) TUNEL staining on liver sections from A20^LPC-KO^ and wild-type (WT) mice 5 h after mouse TNF injection, staining apoptotic cells in red. Scale bar=100μm. (**e**) Serum ALT and AST levels of A20^LPC-KO^ mice (*n*=7) and control (WT; *n*=9) mice 5 h after mTNF injection. (**f**) Serum IL6 levels of A20^LPC-KO^ mice (*n*=2) and control (WT; *n*=3) mice 5 h after mTNF injection. (**g**) Western blot analysis of A20, caspase-3 and actin expression in liver tissue from A20^LPC-KO^ mice and control littermate mice (WT) either or not injected with 10 *μ*g mouse TNF for 5 h. Westerns blots are representative of two independent experiments. (**h**) Caspase activity assayed on liver tissue homogenates of A20^LPC-KO^ mice and WT littermates either or not injected with TNF for 5 h. *,*P*<0.05

Independent from its role as a modulator of NF-*κB* signalling, A20 functions as an anti-apoptotic protein in several cell types, including enterocytes^11^ and hepatocytes.^19^ To investigate whether A20 protects hepatocytes from TNF-mediated apoptosis *in vivo*, we injected mice with a sublethal (10 μg) dose of recombinant mouse TNF. At this dose, TNF normally induces hepatoprotective mechanisms through NF-*κB*-mediated induction of anti-apoptotic genes such as A20 and Bcl-XL, protecting mice from TNF-induced liver toxicity^7^ (Figures 2a and b and Supplementary Fig. S4). In contrast to control mice, which all resist the TNF challenge and only show a modest drop in body temperature, A20^LPC-KO^ mice display severe hypothermia, and were euthanized between 12 and 30 h post-injection for ethical reasons (Figures 3a and b). Histological and microscopic signs of massive liver destruction could be observed in TNF-injected A20^LPC-KO^ mice. The architecture of the liver parenchyma was completely destroyed 6 h post-injection, with clearly visible pyknotic nuclei and numerous red blood cells, in contrast to control liver tissue which looked completely normal (Figure 3c). Liver cell death was also confirmed by TUNEL staining, where many hepatocytes in A20^LPC-KO^ liver tissue stain positive in contrast to very few TUNEL-positive hepatocytes in control tissue (Figure 3d). Massive liver damage upon TNF challenge in A20^LPC-KO^ mice was associated with release of liver-specific enzymes aspartate transaminase (AST) and alanine transaminase (ALT) in the blood (Figure 3e). Also the pro-inflammatory cytokine IL-6 was abundantly present in serum from TNF-injected A20^LPC-KO^ mice, while much less abundant in serum from control littermate mice (Figure 3f). In line with these observations, procaspase-3 processing and caspase-3 activity could be detected in livers of A20^LPC-KO^ mice but not of control mice, as shown by a caspase-3-specific enzymatic DEVD-activity assay (Figures 3g and h). Together, these data identify A20 as an essential protein protecting mice from TNF-induced hepatocyte apoptosis and acute liver failure *in vivo*.

### A20^LPC-KO^ hepatocytes die from TNF-induced FADD-dependent apoptosis

**Figure 4:**
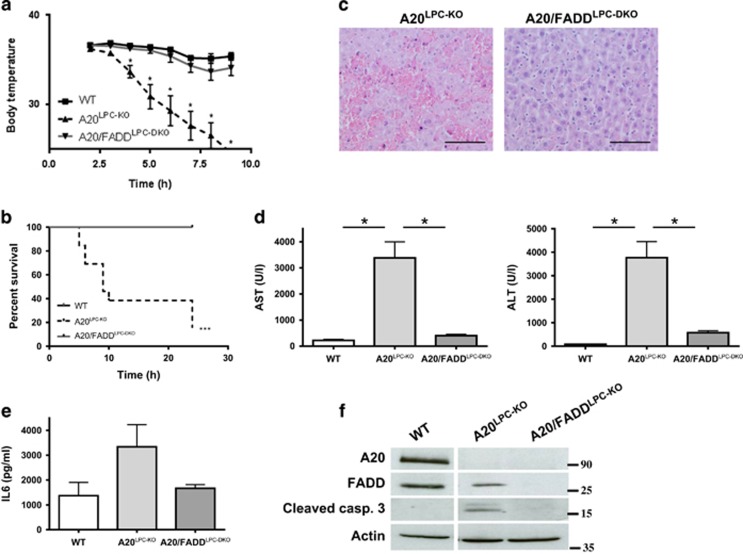
A20^LPC-KO^ hepatocytes die from TNF-induced FADD-dependent apoptosis. (**a-b**) Body temperature (**a**) and survival (**b**) of A20/FADD^LPC-DKO^ (*n*=6), A20^LPC-KO^ mice (*n*=13) and control (WT; *n*=7) littermates after i.p. injection of 10 *μ*g mTNF. *,*P*<0.05; ***,*p*=0.0002 *versus* control. (**c**) Hematoxylin and eosin (H/E) staining on liver sections from A20^LPC-KO^ and A20/FADD^LPC-DKO^ littermate mice injected with TNF for 5 h. Scale bar=100 μm. (**d**) Serum ALT and AST levels of A20/FADD^LPC-DKO^ (*n*=5), A20^LPC-KO^ mice (*n*=9) and control (WT; *n*=10) mice 5 h after mTNF injection. (**e**) Serum IL-6 levels of A20/FADD^LPC-DKO^ (*n*=3), A20^LPC-KO^ mice (*n*=7) and control (WT; *n*=5) mice 5 h after mTNF injection. (**f**) Western blot analysis of A20, FADD, cleaved caspase-3 and actin expression in liver tissue from A20/FADD^LPC-DKO^, A20^LPC-KO^ mice and control littermate mice (WT) 5 h after injection with 10 *μ*g mouse TNF. *,*P*<0.05. ALT, alanine aminotransferase; AST, aspartate aminotransferase; FADD, Fas-associated death domain; TNF, tumor necrosis factor; WT, wild type

In the absence of A20, hepatocytes are hyper-responsive to TNF-induced apoptosis. To confirm the direct cytotoxic effect of TNF inducing death receptor-dependent apoptosis of hepatocytes, we generated A20/FADD^LPC-DKO^ mice by crossing the A20^LPC-KO^ line with mice having a floxed FADD allele (FADD^FL/FL^).^22^ These mice lack both A20 and FADD specifically in liver parenchymal cells (Supplementary Fig. S5). Fas-associated death domain protein is a key adaptor protein signalling to apoptosis from activated death receptors, including TNFR1.^23^ As such, liver-specific FADD-deficient mice (FADD^LPC-KO^) are insensitive to anti-Fas and LPS+galactosamine (GalN)-induced TNFR1-mediated liver toxicity^24, 25^ (Supplementary Fig. S6). To evaluate the contribution of FADD-dependent signalling to the lethal phenotype seen in A20^LPC-KO^ mice injected with TNF, we challenged A20/FADD^LPC-DKO^ mice and A20^LPC-KO^ littermates with a sublethal dose of TNF, as described above. In contrast to A20^LPC-KO^ mice, which all died between 8 and 25 h post-injection, A20/FADD^LPC-DKO^ mice all support this dose of TNF and only show a modest drop in body temperature similar to wild-type control littermate mice (Figures 4a and b). In agreement, liver damage, as detected by histology and serum ALT and AST levels, was strongly reduced in A20/FADD^LPC-DKO^ mice, compared with A20^LPC-KO^ mice (Figures 4c and d). Also IL-6 levels were reduced in A20/FADD^LPC-KO^ mice (Figure 4e). Finally, procaspase-3 processing could only be detected after TNF challenge in the liver of A20^LPC-KO^ mice, not in control or A20/FADD^LPC-DKO^ mice (Figure 4f). These data clearly confirm the essential role of A20 in protecting hepatocytes from FADD-dependent TNF-induced apoptosis.

### Hepatocyte-specific A20 deletion enhances susceptibility to chemically and high fat diet induced hepatocarcinogenesis.

**Figure 5:**
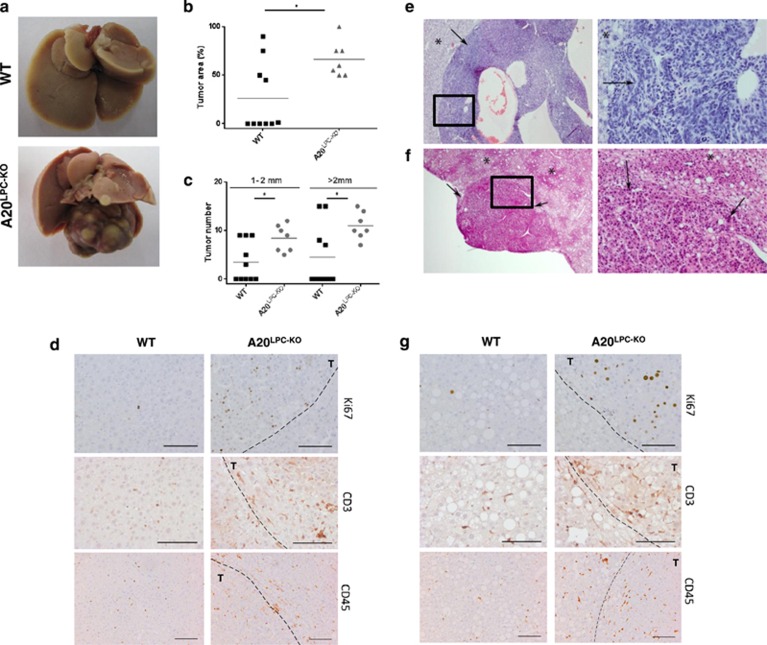
Hepatocyte-specific A20 deletion enhances susceptibility to chemically induced and HFD-induced hepatocarcinogenesis. (**a-d**) Female A20^LPC-KO^ and control littermate mice (WT) were injected with DEN at day P15 and were sacrificed 70 weeks after DEN injection. (**a**) Representative pictures of livers from hepatocyte-specific A20 knockout (A20^LPC-KO^) and control WT littermate mice. (**b**) Percentage of tumor occupied liver area, and (**c**) numbers of tumors size 1-2 mm and>2 mm in livers. *,*P*<0.05. (D) Immunostaining for proliferating cells (Ki67) and infiltrating immune cells (CD3, CD45). Scale bar=100 *μ*m. T, tumor area. (**e-g**) Male A20^LPC-KO^ and control littermate mice (WT) were fed with an HFD for 1 year. Representative picture of a hepatoblastoma (arrows) recorded in HFD-A20^LPC-KO^ mouse (**e**). Representative picture of a hepatocellular adenoma (arrows) recorded in HFD-A20^LPC-KO^ mouse (**f**). Non-neoplastic tissue is depicted by asterisk. The right pictures are a higher magnification to the boxed areas. (**g**) Immunostaining for proliferating cells (Ki67) and infiltrating immune cells (CD3, CD45). Scale bar=100 *μ*m. T, tumor area. DEN, diethylnitrosamin; HFD, high fat diet; WT, wild type

It has become clear that canonical and non-canoncial NF-*κB* signalling has a critical role in cancer development and progression.^26, 27, 28^ NF-*κB* controls pro-survival gene expression and the ability of malignant cells to resist apoptosis-based tumor surveillance mechanisms. Moreover, NF-*κB* also regulates tumor angiogenesis and invasiveness. As a consequence, constitutively active NF-*κB* can be observed in many types of cancer, including hepatocellular cancer (HCC). Since A20^LPC-KO^ mice develop a condition of chronic liver inflammation and are sensitized to hepatocyte apoptosis, we wondered whether A20^LPC-KO^ mice are also sensitized to HCC development. Since these mice do not develop spontaneous HCC (Figure 1), we subjected A20^LPC-KO^ mice and control littermates to the chemical carcinogen diethylnitrosamine (DEN). A single injection of the tumor initiator DEN in 15-day-old C57BL/6 mice induces hepatocyte DNA damage resulting in HCC with a course of development similar to human HCC.^29^ Although female mice are less sensitive than males to DEN-induced hepatocarcinogenesis,^29^ only part of the males displayed typical HCC at the age of 40 weeks, but no significant differences in tumor-occupied liver area or number of macroscopically detectable tumors could be observed between DEN-injected A20^LPC-KO^ and control littermate mice (Supplementary Fig. S7). In contrast, A20^LPC-KO^ female mice developed more and larger tumors and had significantly larger tumor-occupied area compared with wild-type littermates 70 weeks after DEN injection (Figures 5a and c and data not shown). Also hepatocyte proliferation, as assessed by Ki67 staining, and immune cell infiltration was increased in livers from DEN-treated A20^LPC-KO^ mice, compared with control mice (Figure 5d). We next subjected A20^LPC-KO^ and control littermate mice to a high fat diet (HFD). Increased hepatocyte fat uptake by long-term HFD feeding leads to NAFLD that can progress to fibrosis, cirrhosis and even HCC.^30^ Thus, increased fat uptake represents a risk factor for HCC development. In addition pro-inflammatory cytokines such as IL1β, IL-6 and TNF have been shown to promote HFD-induced obesity and tumorigenesis.^31^ A20^LPC-KO^ mice fed on HFD for 20 weeks exhibited a marked increase in the severity of lobular inflammation, ballooning, degeneration and overall NAS (sum of steatosis+ballooning+inflammation) when compared with littermate controls on HFD (Table 2). In mice fed with HFD for 1 year, the severity of lobular inflammation, numbers of inflammatory foci and NAS further increased and was significantly more severe in A20^LPC-KO^ mice compared with the control wild-type group. Moreover, three out of seven HFD- A20^LPC-KO^ mice had multiple benign and malignant hepatic tumors (hepatocellular adenoma, HCC and hepatoblastoma), while no evidence of neoplasia was present in all HFD-treated wild-type mice (Figures 5e and f and Table 2). Also, liver sections from A20^LPC-KO^ mice fed on HFD displayed more hepatocyte proliferation and immune cell infiltration compared with control mice on HFD (Figure 5g).

In conclusion, loss of A20 in hepatocytes increases the susceptibility to DEN- and HFD-induced hepatocarcinogenesis.

## Discussion

To investigate the role of A20 in the adult liver and in conditions of chronic liver inflammation (hepatitis) and hepatocarcinogenesis, we generated mice with hepatocyte-specific deletion of A20. A20^LPC-KO^ mice appear healthy and show no obvious spontaneous pathologies. However, dissection of livers from hepatocyte A20 knockout mice revealed low grade chronic liver inflammation without development of fibrosis or HCCs even at old age. Consistent with the essential role of A20 as a negative feedback regulator of NF-*κB* signalling, A20-deficient liver tissue displayed sustained NF-*κB*-dependent gene expression upon TNF or LPS challenge, demonstrating an important role for A20 in the control of inflammatory reactions in liver.

We previously described A20 as an anti-apoptotic factor in intestinal epithelial cells essential to prevent TNF-induced apoptosis.^11, 32^ Also in hepatocytes, as shown in this study, A20 mainly acts as a protective factor. As a consequence, hepatocyte-specific A20 knockout mice are highly sensitive to a normally sub-lethal dose of TNF, inducing hypothermia and lethality caused by massive hepatocyte apoptosis. In most cell types, TNF-induced cell death requires inhibition of NF-*κB*-induced expression of pro-survival genes. A20-deficient hepatocytes and IECs, however, undergo TNF-induced apoptosis with intact NF-*κB* activity, which suggests that A20 is an essential anti-apoptotic protein induced by NF-*κB* upon TNF stimulation in these cells.

Although the molecular mechanism by which A20 restricts NF-*κB* activation is well characterized,^12^ the mechanism of its anti-apoptotic functions has long been unclear. A20 was identified as part of the death-inducing signalling complex, being recruited after activation of death receptors, where it physically interacts with polyubiquitinated caspase-8.^33^ *In vitro* studies suggested that A20 could probably act as a deubiquitinase, inhibiting caspase-8-induced apoptosis.^33^ A20 has also been shown to increase polyubiquitination of RIP1, enabling RIP1 binding to the caspase-8 protease domain to inhibit its dimerization, cleavage and activation,^34^ and yet another study suggested that A20 blocks TNF-induced apoptosis through suppression of the JNK by targeting apoptosis signal-regulating kinase 1 for proteasomal degradation.^35^ However, very recently, A20 was shown to inhibit cell death by stabilization of linear ubiquitin chains in the death receptor complex, protecting them from cleavage by competing deubiquitinases.^36, 37^

The liver has remarkable regenerative capacity, and surviving hepatocytes massively start proliferating following hepatic loss or injury in order to restore liver function and mass. Mechanistically, it is still unclear if compensatory proliferation following cytotoxic injury differs from liver regeneration after hepatectomy. In different mouse models, however, including the TAK1 and NEMO liver-specific knockout mouse model,^38, 39, 40^ hepatocyte death is the driving force triggering proliferation, favouring hepatocarcinogenesis.^1^ Hepatocyte cell death thus represents a tumor-promoting condition, mediated by compensatory hepatocyte proliferation and inflammation. Since A20 controls both inflammatory and anti-apoptotic activities in the liver, hepatocyte-specific A20 knockout mice are also sensitized to the development of hepatocellular cancer.

Previous studies have identified A20 as a tumour suppressor protein in B cells where its inactivation leads to constitutive or aberrant NF-*κB* activation mediating increased cell proliferation and survival of subsets of B-lineage lymphomas.^41, 42, 43^ Also in the intestine, A20 acts as a tumor suppressor for colon carcinogenesis.^32, 44^ In other cell types, however, A20 has been ascribed pro-tumorigenic activities, likely connected to its anti-apoptotic functions.^12^ These observations suggest that depending on the cell type and tumor stage, A20 may act as a tumor suppressor or a tumor enhancer. In the context of the liver, A20 was found to be preferentially expressed in hepatitis B virus-related HCC cell lines and HCC clinical tissues, contributing to cellular proliferation and survival,^45, 46^ suggesting A20 as a tumor-promoting agent. This is in contrast to a recent study in which A20 was identified as a tumor suppressor in the progression and metastasis of HCC through a mechanism involving inhibition of expression of the EMT-related gene Twist1 via suppression of NF-*κB* activation.^47^ Our *in vivo* findings in mice, however, establish A20 as a crucial protective and tumor suppressive factor in liver, and suggest that A20 deficiency or defects in its activity in humans might sensitize to the development of liver disease and liver cancer in specific contexts. This may be the case in patients bearing specific mutations or polymorphisms in the *A20* locus, making them vulnerable to develop chronic liver diseases such as alcoholic liver disease, NAFLD and viral hepatitis, conditions known to promote the development of cirrhosis and HCC. Moreover, A20 has been identified as a susceptibility gene for autoimmune hepatitis,^13^ a disease which may also lead to liver failure, cirrhosis and HCC.^48^

In conclusion, we here described the dual importance of A20 in hepatocytes. On one hand, A20 negatively regulates NF-*κB* activation and inflammatory signalling. On the other hand, A20 acts as a major cytoprotective protein for hepatocytes in inflammatory and cytotoxic conditions. As a consequence, hepatocyte A20-deficient mice show sustained NF-*κB* signalling, chronic liver inflammation, hepatocyte death and eventually leading to HCC development.

## Materials and Methods

### Generation of tissue-specific A20-deficient mice

Conditional A20/tnfaip3 and FADD knockout mice were generated as described before.^11, 22^ A20^FL/FL^ mice were crossed with Alfp-Cre transgenic mice^20, 21^ to generate a hepatocyte-specific A20 knockout mouse (A20^LPC-KO^). Experiments were performed on mice backcrossed into the C57BL/6 genetic background for at least five generations. Mice were housed in individually ventilated cages in the specific pathogen-free animal facility of the VIB Inflammation Research Center. All experiments on mice were performed according to institutional, national and European animal regulations.

### Liver injury

All experiments were performed on pathogen-free male mice between 8 and 10 weeks of age. *E. coli*-derived recombinant mTNF had a specific activity of 9.46 × 10^7^ IU/mg, and was produced and purified to homogeneity in our laboratory. Endotoxin levels did not exceed 1 ng/mg protein. TNF was administered i.p. at concentrations of 1 *μ*g or 10 *μ*g per 25 g of body weight. Serum ALT and AST levels were measured in the Laboratory of Clinical Biology of the University Hospital Gent, according to standard procedures. For diet studies, mice received irradiated standard diet (10% kcal fat, Research Diets Inc., D12450B) or irradiated HFD (60% kcal fat, Research Diets Inc., D12492) from the age of 8 weeks. For liver carcinogenesis studies, 15-day-old A20^LPC-KO^ mice and control littermates were injected i.p. with 20 mg/kg DEN (Sigma, St. Louis, MO, USA). After 60 weeks, all mice were sacrificed and their livers removed. Percentage tumor area was measured, and externally visible tumors (1-2 mm and>2 mm) were counted by stereomicroscope.

### Western blot analysis

Cells and liver tissue were homogenized using E1A lysis buffer (50 mM HEPES pH7.6; 250 mM NaCl; 5 mM EDTA; 0.5% NP40). Protein lysates were prepared from liver samples, and 40 μg lysates were separated by sodium dodecyl sulphate-polyacrylamide gel electrophoresis, transferred to nitrocellulose and analysed by immunoblotting. Membranes were probed with the following antibodies: anti-A20 (Santa Cruz Biotechnologies, Dallas, TX, USA), anti-caspase-3 (Cell Signaling Technology, Danvers, MA, USA), anti-cleaved caspase-3 (Cell Signaling Technology), JNK (Santa Cruz Biotechnologies), phospho-JNK (Cell Signaling Technology), I*κBα* (Santa Cruz Biotechnologies), phospho-I*κBα* (Cell Signaling Technology), Erk (Cell Signaling Technology), phospho-Erk (Cell Signaling Technology) and anti-actin (Santa Cruz Biotechnologies). As secondary antibodies, anti-rabbit-HRP, anti-mouse-HRP and anti-goat-HRP were used (Amersham Bioscience, Buckinghamshire, UK).

### Apoptosis assays

Apoptosis was analysed by fluorescence microscopy using an *in situ* cell death detection kit (Roche Diagnostics, Basel, Switzerland). Caspase activity was measured by incubation of 25 μg tissue homogenate with 50 *μ*M acetyl-Asp-Glu-Val-Asp-aminomethylcoumarin (Ac-DEVD-amc) (Peptide Institute, Osaka, Japan) in 150 μl cell-free system buffer (10 mM HEPES–NaOH pH 7.4, 220 mM mannitol, 68 mM sucrose, 2 mM NaCl, 2.5 mM KH_2_PO_4_, 0.5 mM EGTA, 2 mM MgCl_2_, 5 mM pyruvate, 0.1 mM PMSF, 1 mM dithiothreitol). The release of fluorescent 7-amino-4-methylcoumarin was measured for 50 min at 2-min intervals by fluorospectrometry at 360 nm excitation and 480 nm emission wavelength, and the maximal rate of increase in fluorescence was calculated (Δfluorescence/min) (Cytofluor; PerSeptive Biosystems, Cambridge, MA).

### Histological analysis

Histology was performed on paraffin-embedded (3 *μ*m) or frozen tissue sections (5 or 10 *μ*m). Briefly, livers were dissected and fixed in 4% paraformaldehyde and embedded in paraffin. Liver sections were examined for the presence of histopathologic lesions and scored as follows: for steatosis (amount of lipid accumulation), grade 0<5% 1=5-33% 2= 33-66% 3=>66% with 0.5 interval; location of steatosis in hepatic lobule, 0= zone 3; 1= zone 1; 2=azonal; 3=panacinar; microvesicular steatosis, 0=not present; 1= present; percentage of hepatocytes affected by microvesicular steatosis (MI%), compared with hepatocytes affected by macrovesicular steatosis; hepatocellular ballooning, 0= none; 1= few balloon cells; 2= many cells/prominent ballooning with 0.5 interval; lobular inflammation, 0= none; 1=<2 foci per 200 × ; 2=2-4 foci; 3=>4 average foci/200 × field; with 0.5 interval; portal inflammation, Pigmented macrophages, Oval cell proliferation, Anisokaryosis, and additional lesions: Scored semi-quantitatively on a scale of 0 to 5. Scores were given as absent (0), subtle (1), mild (2), moderate (3), severe (4) and marked (5) for each criteria with 0.5 interval. The NAFLD activity score (NAS) was calculated as previously described.^49, 50^ Slides were examined unbiased by two board-certified pathologists. Sections (2 μm) of livers (fixed in 4% paraformaldehyde and paraffin-embedded) were stained with hematoxylin/eosin or various antibodies. Incubation in Ventana buffer and staining was performed on a NEXES immunohistochemistry robot (Ventana Instruments) using an IVIEW DAB Detection Kit (Ventana/Roche, Mannheim, Germany) or on a Bond MAX (Leica GmBH, Heidelberg, Germany). CD3, B220, F4/80 and Ki67 antibodies were applied as recently published.^28^ For analysis of fibrosis, slides were stained with Picro sirius red, and three × 10 digital images were selected randomly from every section and images were analysed using in-house developed software to quantify the percentage of fibrosis compared with the total amount of tissue within an image.

### Quantitative real-time PCR

Total RNA from mouse liver samples was isolated and lysed in RNA lysis buffer (Aurum Total RNA Mini kit, Bio-Rad Laboratories, Hercules, CA, USA) on ice for 5 min. One *μ*g RNA was purified using the Aurum Total RNA Mini kit (Bio-Rad Laboratories) and cDNA synthesis was performed using the iScript cDNA synthesis kit (Bio-Rad Laboratories) according to the manufacturer's instructions. 10 ng of cDNA was used for quantitative PCR in a total volume of 10 *μ*l with LightCycler 480 SYBR Green I Master Mix (Roche) on a LightCycler 480 (Roche). Real-time PCR reactions were performed in triplicates. The following mouse-specific primers were used (5′-3′): HPRT forward, AGTGTTGGATACAGGCCAGAC; HPRT reverse, CGTGATTCAAATCCCTGAAGT; A20 forward, AAACCAATGGTGATGGAAACTG; A20 reverse, GTTGTCCCATTCGTCATTCC; I*κBα* forward, GTAACCTACCAAGGCTACTC; I*κBα* reverse, GCCACTTTCCACTTATAATGTC; TNF forward, ACCCTGGTATGAGCCCATATAC; TNF reverse, ACACCCATTCCCTTCACAGAG; IL1β forward, CACCTCACAAGCAGAGCACAAG; IL1β reverse, GCATTAGAAACAGTCCAGCCCATAC; IL-6 forward, GAGGATACCACTCCCAACAGACC; IL-6 reverse, AAGTGCATCATCGTTGTTCATACA; MCP1 forward, GCATCTGCCCTAAGGTCTTCA; MCP1 reverse, TGCTTGAGGTGGTTGTGGAA. Values were normalized to the level of HPRT and GAPDH mRNA, and analysed using qBase+ software (Biogazelle, Gent, Belgium).

### Statistical analysis

Results are expressed as the mean±s.e.m. Statistical significance between experimental groups was assessed using an unpaired two-sample Student *t*-test.

## Figures and Tables

**Table 1:** Average scores of selected lesions in A20^LPC-KO^ mice (KO) and control (WT) littermates

**Group**	**Lesions**					
	**Steatosis**	**Ballooning**	**Lobular inflammation**^a^	**NAS**	**Neoplasia**	**% Fibrotic area**
WT (*n*=5) 20 weeks	0.70	0.12	0.20 (1)	1.00	No	0.35±0.05
KO (*n*=7) 20 weeks	0.71	0.07	0.50 (2)	1.29	No	0.44±0.04
WT (*n*=4) > 1 year	0.12	0	0.12 (0.25)	0.25	No	NA
KO (*n*=2) > 1 year	1.50	0.75	0.5 (2.5)	2.75	No	NA

Abbreviations: KO, knockout; WT, wild type

a Average number of inflammatory foci per 5 × 20 fields. N.A., not analysed

**Table 2:** Average scores of selected lesions in A20^LPC-KO^ mice (KO) and control (WT) littermates

**Group**	**Lesions**				
	**Steatosis**	**Ballooning**	**Lobular inflammation**^a^	**NAS**	**Neoplasia**
HFD-WT (*n*=6) 20 weeks	1.58	0.16	0.91 (4.16)	2.66	No
HFD-KO (*n*=5) 20 weeks	1.70	0.40	1.30 (7.20)	3.40	No
HFD-WT (*n*=4) > 1 year	2.38	1.00	1.00 (5.25)	4.37	No
HFD-KO (*n*=7) > 1 year	2.29	1.07	1.37 (6.43)	4.57	H, HCC, HB

Abbreviations: H: Hepatocellular adenoma; HB, hepatoblastoma; HCC, hepatocellular carcinoma; HFD, high fat diet, KO, knockout, WT, wild type

a Average number of inflammatory foci per 5 × 20 fields

## Abbreviations

A20^LPC-KO^: liver parenchymal cell-specific A20 knockout
A20/FADD^LPC-DKO^: liver parenchymal cell-specific A20 FADD double knockout
ALT: alanine aminotransferase
AST: aspartate aminotransferase
DEN: diethylnitrosamin
FADD: Fas-associated death domain
HCC: hepatocellular carcinoma
HFD: high fat diet
LPS: lipopolysaccharide
NF-*κB*: nuclear factor-*κB*
NAFLD: non-alcoholic fatty liver disease
TNFAIP3: Tumor necrosis factor alpha induced protein 3
TNFR: tumor necrosis factor receptor
