# Supplementary material for: A20 prevents chronic liver inflammation and cancer by protecting hepatocytes from death
Source: Cell Death Dis. 2016 Jun 2;7(6):e2250–. doi: 10.1038/cddis.2016.154 (PMC5143384; doi:10.1038/cddis.2016.154)
Supplement: Supplementary Figures [file cddis2016154x1.ppt]

## Slide 1
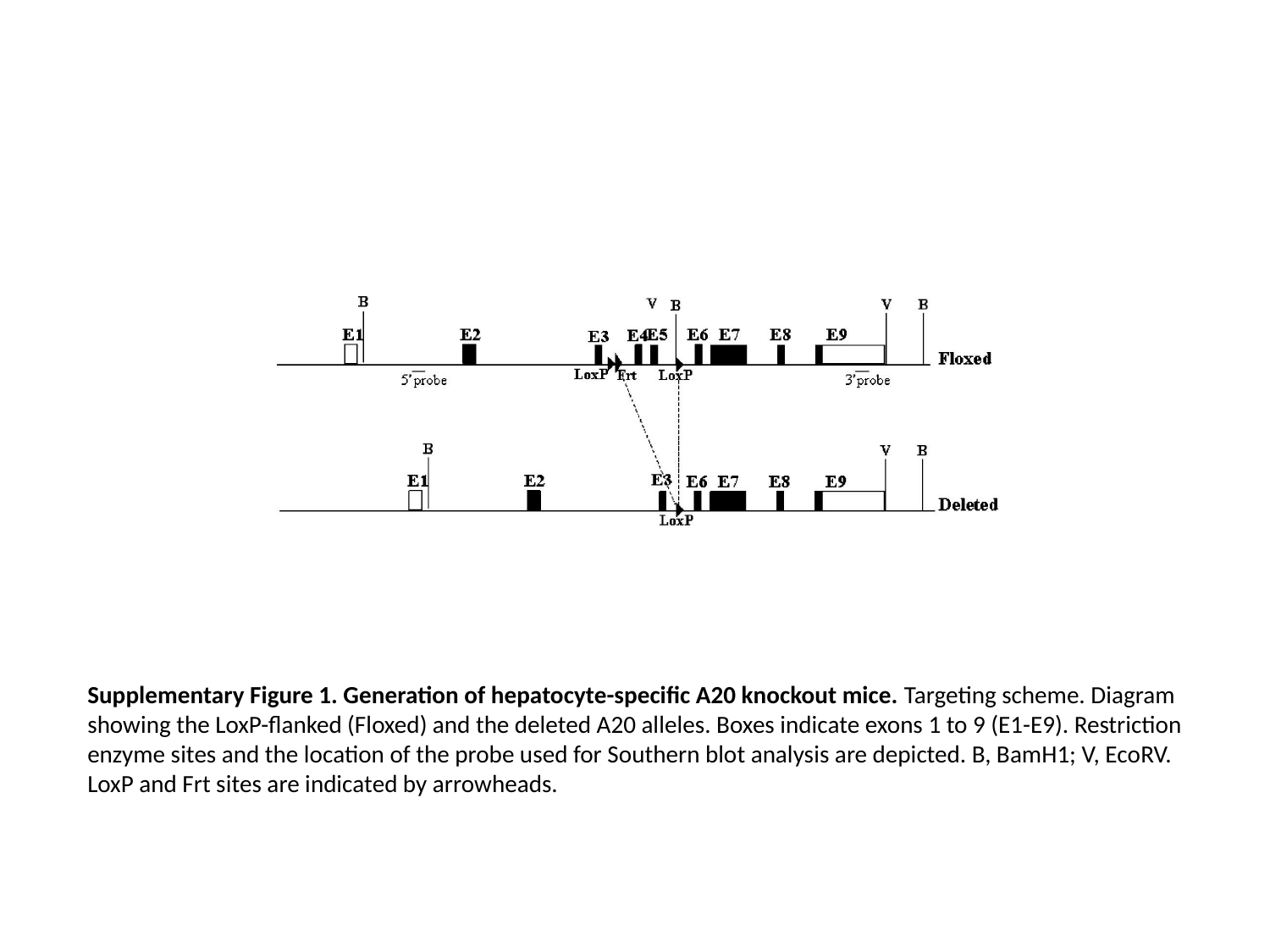

Supplementary Figure 1. Generation of hepatocyte-specific A20 knockout mice. Targeting scheme. Diagram showing the LoxP-flanked (Floxed) and the deleted A20 alleles. Boxes indicate exons 1 to 9 (E1-E9). Restriction enzyme sites and the location of the probe used for Southern blot analysis are depicted. B, BamH1; V, EcoRV. LoxP and Frt sites are indicated by arrowheads.

## Slide 2
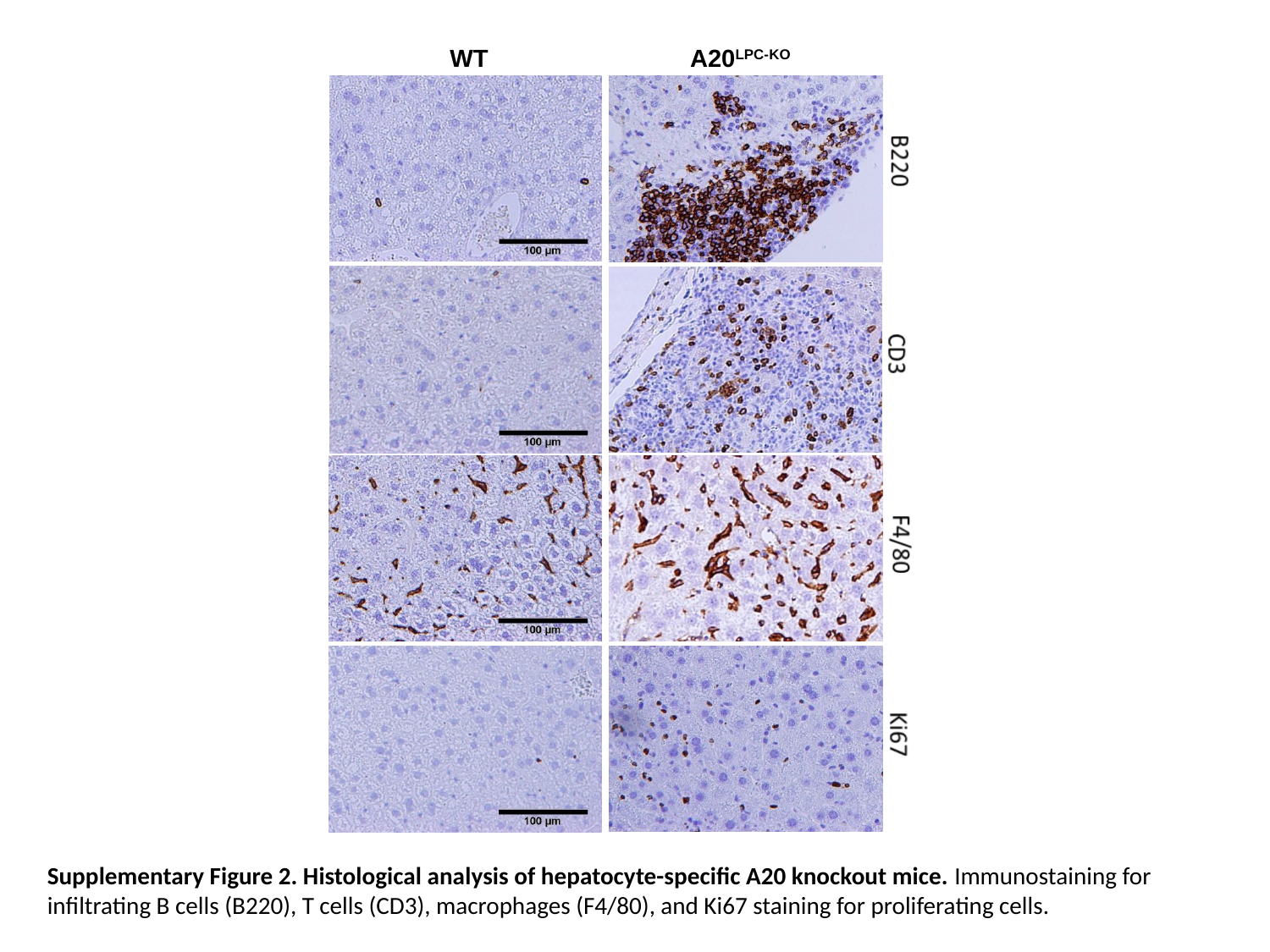

WT
A20LPC-KO
Supplementary Figure 2. Histological analysis of hepatocyte-specific A20 knockout mice. Immunostaining for infiltrating B cells (B220), T cells (CD3), macrophages (F4/80), and Ki67 staining for proliferating cells.

## Slide 3
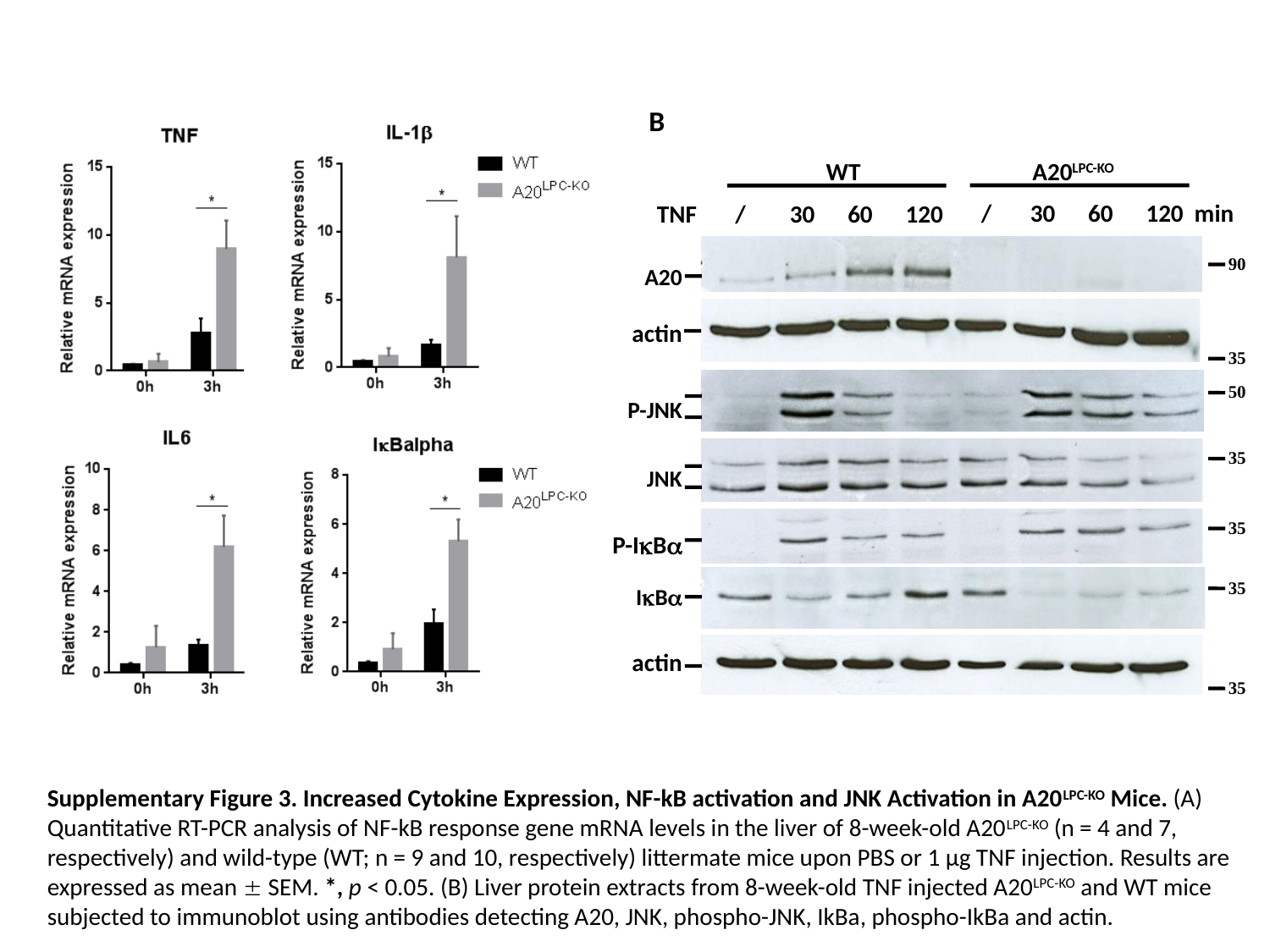

A
B
WT
A20LPC-KO
 / 30 60 120 min
 TNF / 30 60 120
90
A20
actin
35
50
P-JNK
35
JNK
35
35
IκBα
actin
35
P-IκBα
Supplementary Figure 3. Increased Cytokine Expression, NF-kB activation and JNK Activation in A20LPC-KO Mice. (A) Quantitative RT-PCR analysis of NF-kB response gene mRNA levels in the liver of 8-week-old A20LPC-KO (n = 4 and 7, respectively) and wild-type (WT; n = 9 and 10, respectively) littermate mice upon PBS or 1 µg TNF injection. Results are expressed as mean ± SEM. *, p < 0.05. (B) Liver protein extracts from 8-week-old TNF injected A20LPC-KO and WT mice subjected to immunoblot using antibodies detecting A20, JNK, phospho-JNK, IkBa, phospho-IkBa and actin.

## Slide 4
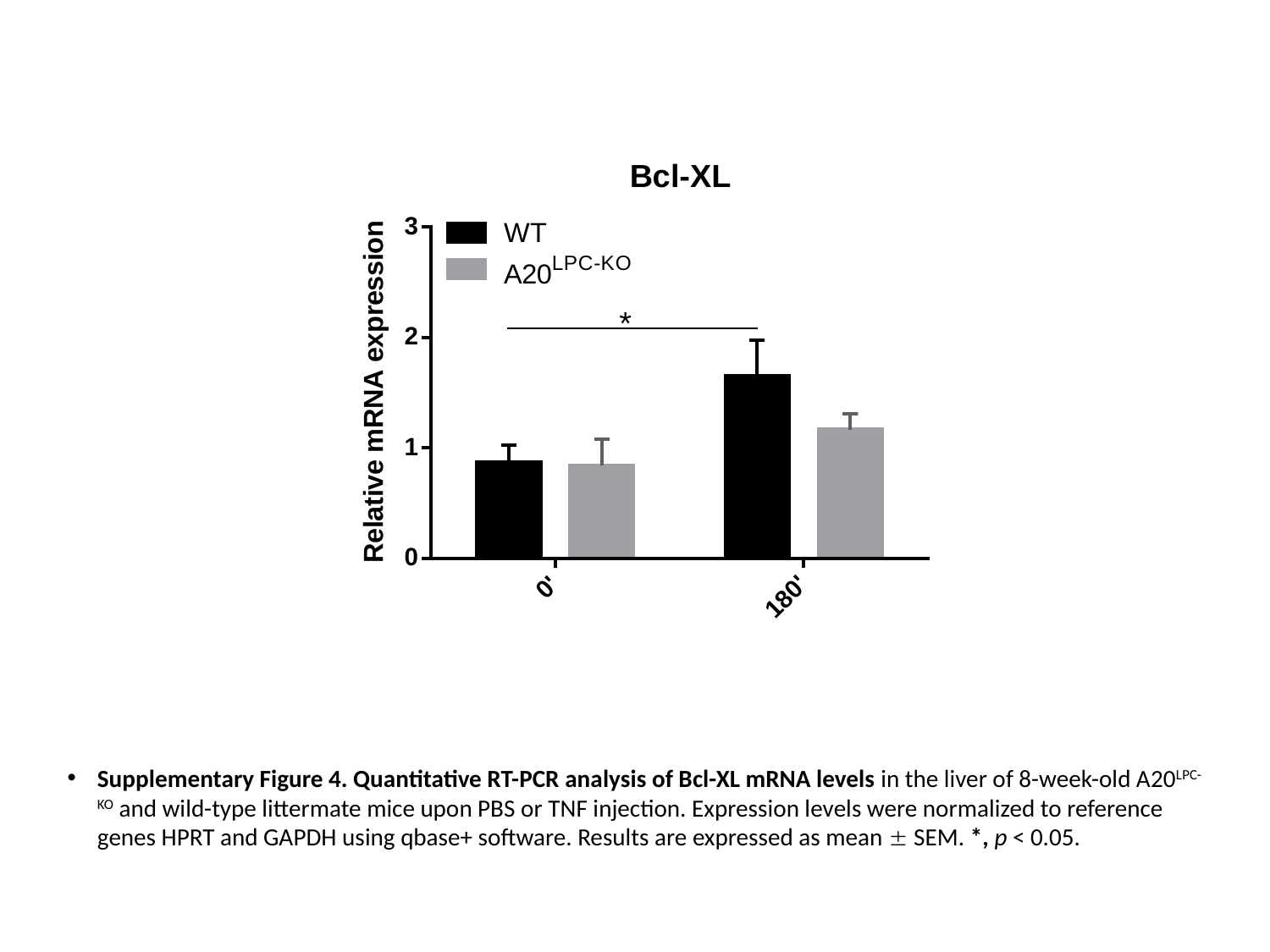

Supplementary Figure 4. Quantitative RT-PCR analysis of Bcl-XL mRNA levels in the liver of 8-week-old A20LPC-KO and wild-type littermate mice upon PBS or TNF injection. Expression levels were normalized to reference genes HPRT and GAPDH using qbase+ software. Results are expressed as mean ± SEM. *, p < 0.05.

## Slide 5
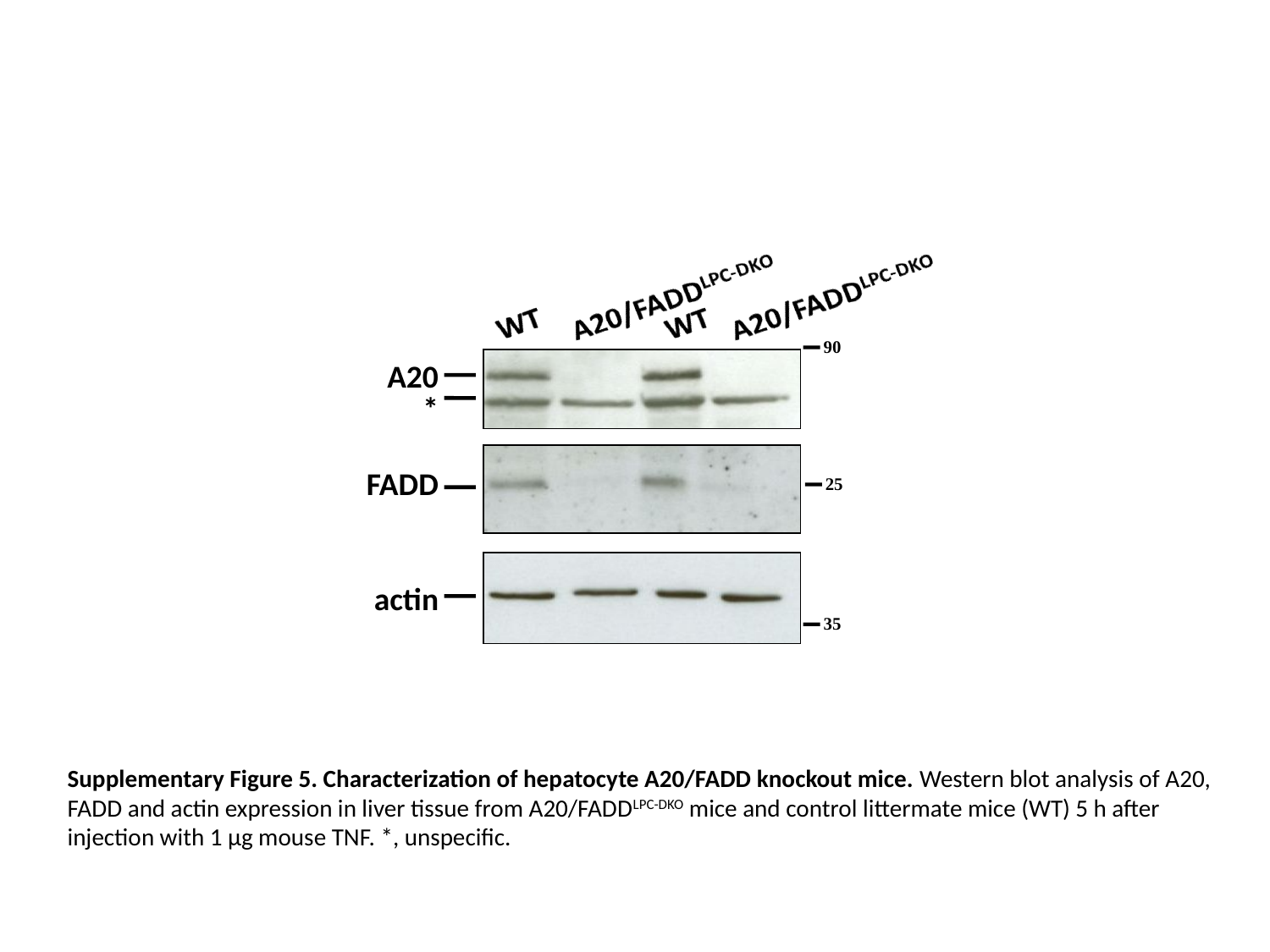

A20
*
FADD
actin
90
25
35
Supplementary Figure 5. Characterization of hepatocyte A20/FADD knockout mice. Western blot analysis of A20, FADD and actin expression in liver tissue from A20/FADDLPC-DKO mice and control littermate mice (WT) 5 h after injection with 1 µg mouse TNF. *, unspecific.

## Slide 6
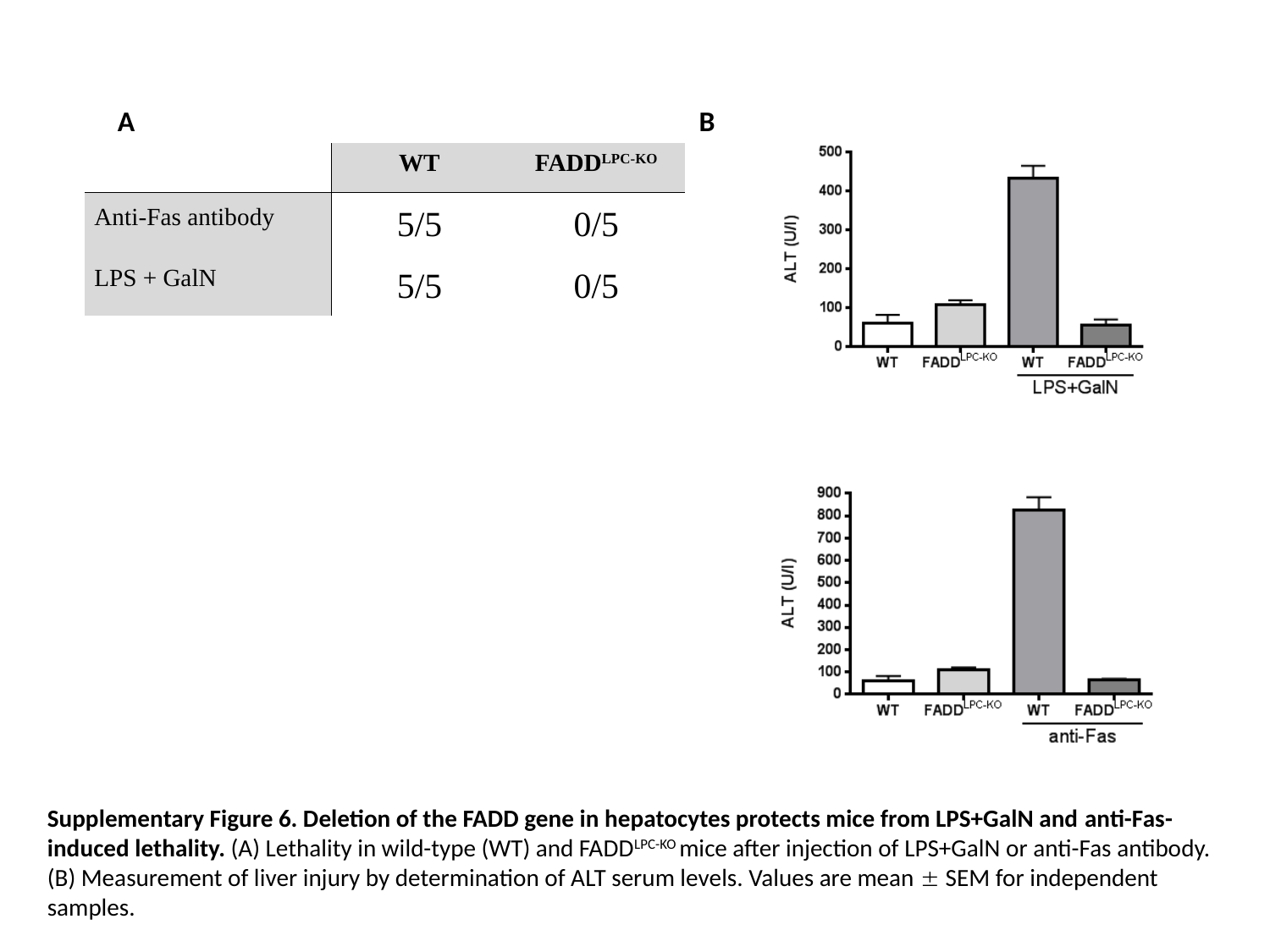

A
B
| | WT | FADDLPC-KO |
| --- | --- | --- |
| Anti-Fas antibody | 5/5 | 0/5 |
| LPS + GalN | 5/5 | 0/5 |
Supplementary Figure 6. Deletion of the FADD gene in hepatocytes protects mice from LPS+GalN and anti-Fas-induced lethality. (A) Lethality in wild-type (WT) and FADDLPC-KO mice after injection of LPS+GalN or anti-Fas antibody. (B) Measurement of liver injury by determination of ALT serum levels. Values are mean ± SEM for independent samples.

## Slide 7
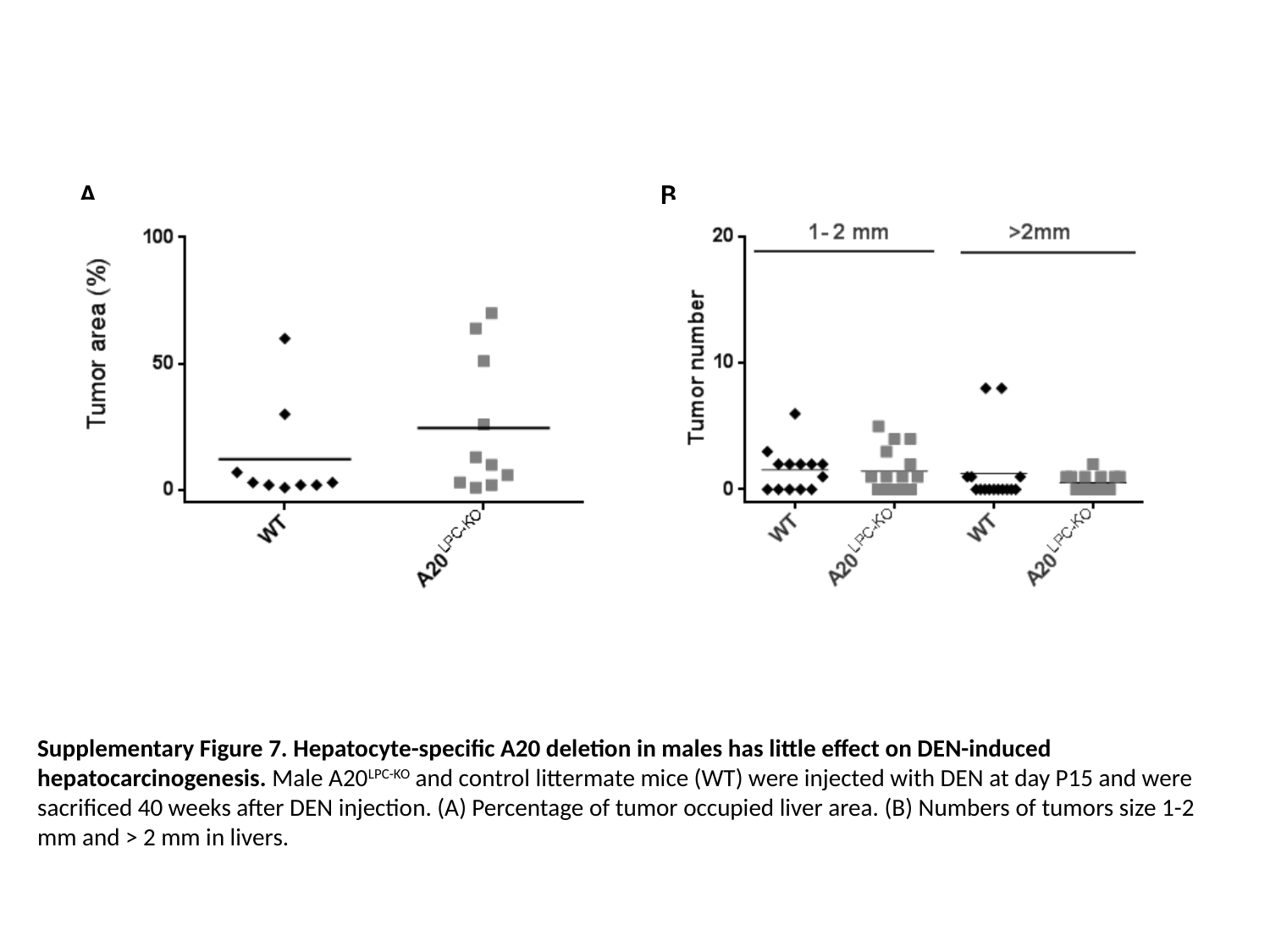

A
B
Supplementary Figure 7. Hepatocyte-specific A20 deletion in males has little effect on DEN-induced hepatocarcinogenesis. Male A20LPC-KO and control littermate mice (WT) were injected with DEN at day P15 and were sacrificed 40 weeks after DEN injection. (A) Percentage of tumor occupied liver area. (B) Numbers of tumors size 1-2 mm and > 2 mm in livers.
